# Incidence, Onset Time and Predictors of Postdural Puncture Headache Among Mothers Who Underwent Caesarean Section in Hospitals, Ethiopia: A Prospective Cohort Study

**DOI:** 10.1155/anrp/5645773

**Published:** 2026-08-24

**Authors:** Esubalew Tesfahun, Eden Takele, Dereje Andargie, Solomon Hailemeskel

**Affiliations:** ^1^ School of Public Health, Asrat Woldeyes Health Science Campus, Debre Berhan University, Debre Birhan, Ethiopia, dbu.edu.et; ^2^ College of Education, Debre Berhan University, Debre Birhan, Ethiopia, dbu.edu.et; ^3^ School of Nursing and Midwifery, Asrat Woldeyes Health Science Campus, Debre Berhan University, Debre Birhan, Ethiopia, dbu.edu.et

## Abstract

**Background:**

Postdural puncture headache (PDPH) is a significant complication of spinal anaesthesia, especially in obstetric patients. It is triggered by the leakage of cerebrospinal fluid (CSF) through the puncture in the Dura mater. Understanding the timing of PDPH onset is crucial for early diagnosis and treatment, but previous studies have not fully explored its timing and associated factors.

**Aim:**

To determine the incidence and predictors of PDPH in women who deliver by caesarean section under spinal anaesthesia.

**Methods:**

A prospective follow‐up study was conducted from March 7 to May 8, 2025, among 365 mothers who underwent caesarean section under spinal anaesthesia at government hospitals found in Debre Berhan, Ethiopia. Data were collected using a structured interviewer‐administered questionnaire. The Kaplan–Meier method was used to estimate the survival time for PDPH, with the log‐rank test for curve comparison. Cox regression analysis was conducted to identify predictor variables.

**Results:**

The cumulative incidence of PDPH was 33.7% (95% CI: 28.9%, 38.7%), and incidence density was 3.75 (95% CI: 3.11, 4.47) per 1000 person‐hours of observation. The mean time to onset of PDPH was 28.9 h (approximately 1.4 days). The restricted mean survival time was 28.98 Hr (95% CI: 25.78, 32.17). Multiple attempts during the procedure AHR 1.69 (95% CI: 1.104, 2.606; *p* < 0.01), larger needle size AHR 8.22 (95% CI: 4.94, 13.67; *p* < 0.001) and anaesthetist having less than 3 years of experience AHR 2.7 (95% CI 1.62, 4.51; *p* < 0.001) were found to be predictors of PDPH.

**Conclusion:**

This study confirmed that reducing the magnitude of PDPH requires limiting the number of puncture attempts and usage of Quincke spinal needles (22G) or fine‐gauge needles (24G‐26G).

## 1. Introduction

A caesarean section (CS) is a surgical procedure that can be crucial for the health and safety of both mothers and babies when complications arise during pregnancy or labor [1]. Advances in anaesthesia and surgical techniques have led to improved outcomes and safety, contributing to an increase in its rates. However, CS carries risks of complications that can lead to morbidity and, in some cases, mortality. Health systems must ensure that access to CS is timely and available when medically necessary, while avoiding unnecessary procedures [2, 3].

Postdural puncture headache (PDPH) is linked to complications like subdural hematoma and chronic headaches, with higher prevalence in obstetric patients [4]. Neuraxial anaesthesia (NA) is most used for lower abdominal and lower extremity surgery. Spinal anaesthesia (SA) is one of the neuraxial techniques where local anaesthetic is placed directly in the intrathecal (subarachnoid) space. SA is the preferred method for CS delivery because of its safety for the mother, simplicity of the technique, reduced maternal risks, and the opportunity it provides for immediate bonding between mother and newborn [5]. However, it is an invasive technique that may result in several problems such as total spinal cord injury, cardiovascular collapse, meningitis, paralysis and PDPH and even death [6, 7].

PDPH associated with SA is more prevalent in obstetric patients than in nonobstetric patients. This becomes especially important for postpartum patients caring for the new‐born child. Complications from PDPH include subdural hematoma, cerebral venous sinus thrombosis, depression, cranial nerve dysfunction, chronic headache and backache. Therefore, understanding the onset time of PDPH is crucial for early diagnosis and differentiation from other types of headaches during the postpartum period. Prompt recognition allows for timely treatment and helps prevent potential complications. This study aims to assess the incidence, time to development and predictors of PDPH.

## 2. Methods and Materials

### 2.1. Study Area and Setting

The study was conducted in Debre Berhan Metropolitan City, Ethiopia. In the town there are 2 public hospitals, one private hospital and 8 health centres. Among the healthcare facilities in the town, one health centre, one private primary hospital, and two public hospitals are performing CS. Each year, approximately 3661 deliveries occur [8].

### 2.2. Study Design and Period

An institution‐based prospective follow‐up study was conducted, and mothers who gave birth with CS under SA were followed up postoperatively from March 7 to May 8, 2025.

### 2.3. Source, Study Population, Sampling Techniques and Procedures

**FIGURE 1:**
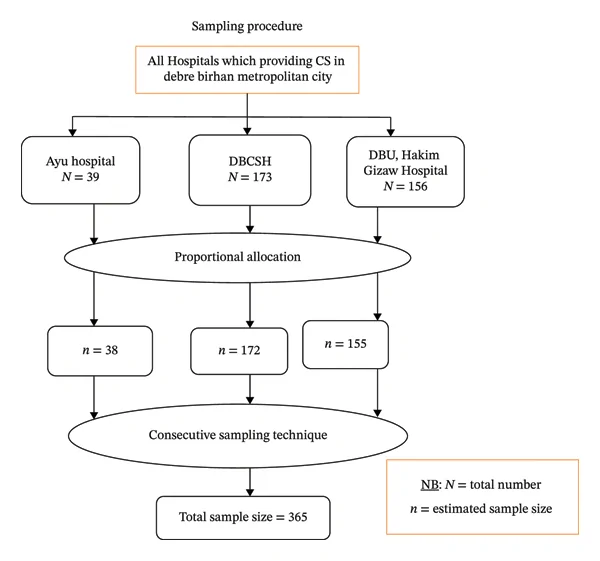
Sampling procedure and proportional allocation for incidence and time to development of PDPH, in Debre Berhan Metropolitan City Hospitals, 2025.

All women who delivered through CS under SA in Hospitals at Debre Berhan Metropolitan City were a source population. All women who delivered through CS under SA during the study period were a study population. The study had included all Hospitals in Debre Berhan Metropolitan City that provide CS services. Sampling allocation was based on the 2‐month average number of women who delivered via CS with SA at each facility, which were approximately 368 women. The total sample size was 365 participants, and a consecutive sampling technique was employed until this sample size is achieved (Figure 1).

### 2.4. Eligibility Criteria

#### 2.4.1. Inclusion Criteria

Women undergoing either elective or emergency CS under SA with ASA Scores II to III was included in the study.

#### 2.4.2. Exclusion Criteria

Women with a pre‐existing diagnosis of chronic headaches or migraines—that condition may complicate the interpretation of the occurrence and symptoms of PDPH—were excluded from this study.

### 2.5. Operational Definition

 
**Diagnosis of PDPH:** Patients were considered as having PDPH if a headache that occurs within 5 days of dural puncture characterized by dull bilateral pain originating in the frontal area and radiate to occiput that will worsen within 15 min of sitting/standing position, sneezing, coughing straining and improves within 15 min of lying down [9].
 
**Survival time:** Survival time in hours is measured from the immediate postoperative time of the patient until a patient develops PDPH until POD five [10].
 
**Start time:** Immediate postoperative day started when she transferred to recovery room.
 
**End time:** Postoperative day five.
 
**Event:** Patients who developed PDPH assumed as event [10].
 
**Censoring:** patients referred to other hospitals, patients who do not develop PDPH until postoperative day five and patients who refused to participate during the follow‐up [10].
 
**Lost follow-up:** Nonresponse patients after five calls and patients whose phone did not work
 
**American Society of Anaesthesiologists (ASA) score** is a classification system used to measure a patient’s preoperative physical condition.
 
**ASA Class I:** A normally healthy patient.
 
**ASA Class II:** A patient with mild systemic disease.
 
**ASA Class III:** A patient with severe systemic disease that is not incapacitating.
 
**ASA Class IV:** A patient with an incapacitating systemic disease that is a constant threat to life.
 
**ASA Class V:** A moribund patient who is not expected to survive for 24 h with or without the operation [11].
 
**Emergency CS:** A CS done once mothers are already in labour, or they may need to have the operation urgently when they are not in labor.
 
**Elective CS:** A procedure to deliver a baby by a surgical procedure and it is planned before the mother goes into labour.

### 2.6. Data Collection Tools, Procedure and Follow‐Up

Data were collected using the Kobo Toolbox by four trained BSc midwife using a pretested questionnaire. Information on sociodemographic characteristics and health behaviours was obtained directly from the patients using a questionnaire. Intraoperative data like the time of operation, duration of surgery, needle size, number of attempts, ASA class and the profession and education level of the anaesthetist in the operating room have been collected from the patient’s operation notes by data extraction checklist. Patients have been followed up daily after the operation until the patients were discharged from the hospital.

After a client was discharged from the hospital, the second phase of data collection commenced using a questionnaire. This phase has been continued until the patient either develops PDPH or was censored (i.e., referral to other hospitals, not developing PDPH within 5 days postoperation [PDPH typically presents 24–72 h postprocedure as a postural headache], loss to follow‐up or withdrawal from the study).

All patients were asked to provide one or more mobile phone numbers or other contact information, including personal phones and those of their attendants, for communication after discharge. Upon discharge, all patients and attendants were receiving information and clear instructions about the characteristics of PDPH to encourage active reporting of any headaches. Patients discharged before five postoperative days without developing PDPH were informed that they will receive follow‐up calls to check on the presence or absence of headaches. A total of five calls were made before a patient is classified as a nonresponder.

### 2.7. Data Processing and Analysis

Once the in‐patient and postdischarge data collection forms are completed, the data has been exported to Stata (V. 14.0) from kobo toolbox for analysis. A descriptive analysis of categorical variables had been conducted using frequency tables, while continuous variables had been summarised with means and standard deviations (SD). The survival time for PDPH will be estimated using the Kaplan–Meier (KM) method. The log‐rank test had been used to compare the estimated survival curves based on categorical variables. The incidence density (rate) and cumulative incidence density were calculated using Stata 14.

Before running the Cox proportional hazards regression model to assess the simultaneous association between multiple covariates and survival, multicollinearity had been examined using the variance inflation factor (VIF) for continuous variables. All variables have been considered acceptable if their VIF values are below 10. The proportional hazards assumption (PHA) had been tested to assess the combined effects of several covariates on the hazard ratio, using the scaled Schoenfeld residuals test (phtest). All variables had been found to satisfy this assumption, with *p* values greater than 0.05.

To identify potential predictors of the time to the development of PDPH, a bi‐variable Cox proportional hazards regression model had been fitted for each explanatory variable. Variables with a *p* value less than 0.25 had been included in the multiple Cox regression model. In the multiple Cox analysis, predictors with a *p* value less than 0.05 had been considered statistically significant for PDPH. The adjusted hazard ratio (AHR) had been used to assess the strength of the association between each predictor and the outcome. The primary outcome of this study was the incidence and predictor of postdural puncture. The secondary outcome measured was median survival time of PDPH.

### 2.8. Ethical Approval and Consent to Participate

Ethical clearance has been obtained from DBU, Asrat Woldeyes Health Science Campus Ethical Review Committee (Ref. No: IRB 01/96/2017 and protocol number IRB‐413). A formal letter of support from the Asrat Woldeyes Health Science Campus, Debre Berhan University, was submitted to each hospital, and permission has been sought. Patient confidentiality has been strictly maintained throughout the research process. If the mother developed a PDPH as an outpatient, she was instructed to visit the nearest clinic for evaluation and treatment. However, if she was an outpatient, the data collectors connected her with a physician for evaluation and treatment based on the severity. The study objectives were oriented to the study participants, and their oral informed consent was obtained from all the study participants before asking the questions.

## 3. Results

### 3.1. Sociodemographic Characteristics

A total of 365 patients were followed; the mean age of the study participants was 27.2 ± 4.3 years. Seeing participant residents, about 332 (91%) were urban dwellers. From 365 mothers, 135 (37%) were government employees; regarding educational status, 237 (64.9%) of participants were college and above achievers (Table 1).

**TABLE 1:** Sociodemographic characteristics of the study participants.

Variable	Category	Frequency	Percent (%)
Age	≤ 25	106	29.0
	26–35	237	64.9
	> 35	22	6.0

Resident	Urban	332	91
	Rural	33	9

Educational status	Unable to read and write	4	1.1
	Able to read and write	26	7.1
	Primary school	22	6.0
	Secondary school	76	20.8
	College and above	237	64.9

Occupational status	Privet employee	105	28.8
	Government employee	135	37.0
	Merchant	45	12.3
	Student	9	2.5
	Housewife	59	16.2
	Unemployed	2	0.5

### 3.2. Patient Related Factors

In this study, the mean body mass index (BMI) was 25.96 ± 3.91 kg/m^2^, with 102 (27.9%) of parturients having a BMI within the normal range (18.5–24.9 kg/m^2^). Regarding previous history of SA, 82.5% of all respondents had no previous history of SA, while 73 (20%) respondents had previous history of SA from these 30 (41.09%) mothers had history of PDPH (Table 2).

**TABLE 2:** Patient related factors for mother having caesarean section under SA.

Variable	Category	Frequency	Percent
BMI	Under weight (< 18.5 kg)	14	3.8
	Normal weight (18.5–24.9 kg)	126	34.5
	Overweight (25–25.9 kg)	202	55.3
	Obese ≥ 30 kg	23	6.3

Gravidity	Primigravida	128	35.1
	Multigravida	237	64.9

ASA	ASAII	358	98.1
	ASAIII	7	1.9

Previous history of SA	Yes	73	20.0
	No	292	80.0

Previous history of PDPH	Yes	30	8.2
	No	335	91.78

### 3.3. Anaesthesia Related Factors

In this study almost all mothers 99.7%, had been given SA in a sitting position. The most frequently used needle sizes were 25 gauge (36.2%) and 24‐gauge needle (35.3%). With respect to needle direction during SA administration, 98.9% were cephalic. Around 63.8% of SA was administered after a single attempt. Regarding the level of education of anaesthetist 245 (67.1%) were at the BSC level. Almost half (55.9%) of the mothers received SA from anaesthetists with four to 6 years of experience (Table 3).

**TABLE 3:** Anaesthesia related factors.

Variable	Category	Frequency	Percent (%)
Needle size	22 Gauge	19	5.2
	23 Gauge	50	13.7
	24 Gauge	129	35.3
	25 Gauge	132	36.2
	26 Gauge	35	9.6

No of attempt	Single attempt	233	63.8
	Twice attempt	102	27.9
	> 2 attempt	30	8.2

Experience of anaesthetist	Student	17	4.7
	1–3 years	45	12.3
	4–6 years	204	55.9
	> 6 years	99	27.1

Level of education of anaesthetist	BSC	245	67.1
	MSC	120	32.9

### 3.4. Incidence of PDPH

Parturients have been followed up for a total of 32,732 person‐hours. Among the mothers who had CS under SA followed, 242 (62.5%) were censored, while 123 (33.7%) developed PDPH during the follow‐up period, and that makes the cumulative incidence 33.7% (95% CI: 28.9%–38.7%). The PDPH incidence density in this study was 3.75 (95% CI: 3.11–4.47) per 1000 person‐hours of observation.

### 3.5. Median Survival Time of PDPH From Date of CS to 5^th^ POD

The overall median survival time was undetermined because the longest observed analysis time was censored. However, the median survival time to the development of PDPH was assessed for different variable categories. For mothers who underwent more than two attempts during SA administration, the median survival time was 18 h. When SA was administered using a larger needle, the median survival time to PDPH was 16 h. Additionally, for mothers who received SA from an anaesthetist with less than 3 years of experience, the median survival time was 18 h.

Since the largest observed analysis time was censored, the mean survival time restricted to the longest follow‐up time was computed instead of the overall median survival time. The restricted mean survival time was 28.98 h (95% CI: 25.78–32.17).

**FIGURE 2:**
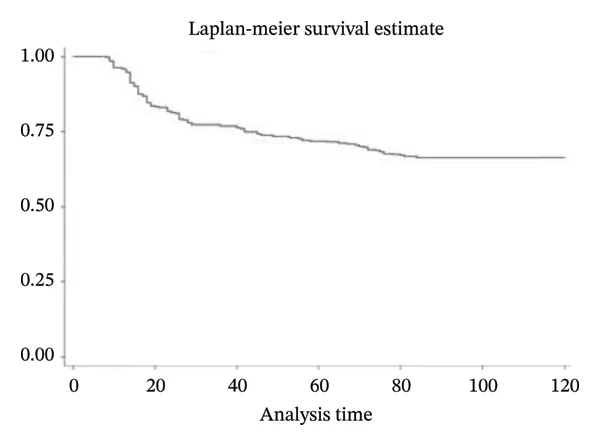
Overall Kaplan–Meier survival estimate of PDPH for mothers after caesarean section under spinal anaesthesia.

In the study, no events occurred within the first 8 hours. Approximately 12.2% of PDPH cases occurred within 12 h after surgery, 55.3% occurred in the following 24 h postoperatively, and more than seven‐tenths of PDPH cases (78.04%) occurred within 48 h after CS (Figure 2).

### 3.6. Predictors of Time to Development of PDPH

The relationship between the baseline variables and the risk of PDPH was analysed using the simple Cox proportional hazard regression model. The results of bi‐variable Cox regression analysis showed that 9 factors were selected for multivariable Cox regression analysis at a *p*‐value < 0.25. In multiple Cox regression analysis, number of attempts, needle size and experience of the anaesthetist were found to be independent predictors of PDPH at *p* value < 0.05.

Multiple attempts of SA (often due to technical difficulties in accessing the subarachnoid space) were 1.69‐fold increase in the hazard of developing PDPH compared to a single attempt AHR: 1.69 (95% CI: 1.10,2.61; *p* < 0.01), larger needle size (22G or 23G) 8.22‐fold higher hazard of developing PDPH compared to smaller needles (24G, 25G or 26G) AHR: 8.22 (95% CI: 4.94,13.67; *p* < 0.001), and SA administered by anaesthetists with less than 3 years of experience were a 2.7‐fold higher hazard of developing PDPH at any given time, compared to procedures performed by anaesthetists having greater than 3 years of experience AHR 2.7 (95% CI: 1.62,4.51; *p* < 0.001) (Table 4).

**TABLE 4:** Multivariable cox regression analysis results of predictors of postdural puncture headache among mothers who underwent caesarean section under spinal anaesthesia.

Predictors	Category	Status		CHR (95% CI)	AHR (95% CI)	*p*‐value
		PDPH	Censored			
Level of education	BSc	96	149	1	1	0.89
	MSc	27	93	0.5 (0.33–0.77)	1.03 (0 0.62–1.7)	

Previous history of SA	Yes	37	36	1	1	0.44
	No	86	206	0.45 (0.30–0.66)	0.68 (0.26–1.78)	

Previous history of PDPH	No	107	228	1	1	0.49
	Yes	16	14	2.15 (1.27–3.64)	1.27 (0.63–2.55)	

Urgency of surgery	Emergency	91	212	1	1	0.84
	Elective	32	30	2.18 (1.46–3.27)	0.91 (0.37–2.20)	

Fluid administered	1000–2000 mL	118	214	1	1	0.07
	2100–3000 mL	5	25	0.36 (0.15–0.89)	0.43 (0.17–1.08)	

Needle direction	Cephalic	119	214	1	1	0.16
	Lateral	4	0	7.4 (2.67–20.5)	2.11 (0.74–6.01)	

Experience of anaesthetist	< 3 years	54	8	8.75 (6.03–12.71)	2.7 (1.62–4.51)	0.001^∗^
	> 3 years	69	234	1	1	

Number of attempts	Single attempt	47	186	1	1	0.01^∗^
	Multiple attempts	76	56	4.04 (2.80–5.83)	1.69 (1.10–2.61)	

Needle size	Larger needle	65	4	16.1 (10.9–23.8)	8.22 (4.94–13.67)	0.001^∗^
	Smaller needle	58	238	1	1	

^∗^Indicates independent variable significantly associated with the occurrence of PDPH.

## 4. Discussion

The cumulative incidence observed in the current study was 33.7% (95% CI: 28.9%–38.7%). This finding is consistent with studies conducted in Kuwait (29.5%) [26], Egypt (32.5%) [12], and Gondar (31.3%) [13]. However, the cumulative incidence observed in the current study (33.7%) is higher than in studies done in Ethiopia, such as the study conducted in Debre Tabor General Hospital (20.2%) [14] and Dilla Hospital (25.7%) [15]; this is due to the difference in length of follow‐up, study design, and maternal hydration status before the procedure. It also exceeds the rates reported in several African countries, such as Ghana (8.3%) [27], and Kenya (20.35) [16] as well as the studies done in India (11.4%) [17] and Jordan (6.3%) [18]. The observed increase in incidence is attributed to differences in demographic characteristics and study design, as well as variations in clinical protocols and supervision levels during the procedures. On the other hand, the incidence found in this study is lower than that reported in Felege Hiwot Hospital, Ethiopia (42.6%). In Felege Hiwot Hospital, larger‐gauge spinal needles (20G, 21G and 22G) were commonly used to facilitate easier cerebrospinal fluid (CSF) flow [19]. This difference in needle size partly explains the higher incidence of PDPH observed in those studies.

The mean time to onset of PDPH was 28.9 h (approximately 1.4 days), which is consistent with findings from studies conducted in Kuwait, and India, reporting mean times of 1.7 days and 1.26 days, respectively [17, 20]. This is due to the physiological response to dural puncture, which typically leads to CSF leakage and a subsequent drop in intracranial pressure within a predictable time frame.

The hazard of developing PDPH was 1.69 times higher for mothers who received SA after multiple attempts compared to a single attempt. This finding is supported by previous studies conducted in Cuba, Jordan, Gondar, Bahir Dar and Dilla, Ethiopia [13, 15, 18, 19, 21]. The increased risk associated with persistent failure to successfully puncture the dura, resulting in continuous CSF leakage and contributing to PDPH. However, a study conducted in India found no significant association between the number of attempts and PDPH. This difference is explained by needle size variations, as that study exclusively used 26‐gauge needles and reported successful SA on the first attempt in 83.5% of parturients. Additionally, only 20% of mothers who required more than two attempts developed PDPH.

The hazard of developing PDPH was 8.2 times higher among mothers who received SA using larger‐gauge needles compared to those who received smaller‐gauge needles. This finding aligns with studies conducted in Cuba, Gondar, Dilla, and Bahir Dar, which also reported an increased risk of PDPH associated with larger needle sizes [13, 15]. The most likely explanation is that larger‐gauge needles create a larger Dural tear, resulting in greater CSF leakage, the primary mechanism behind PDPH. In contrast, smaller‐gauge needles cause less Dural trauma and minimise CSF loss, thereby reducing the risk of PDPH. Larger gauge needles are often chosen for their ability to provide a faster and more reliable flow of CSF, which helps confirm proper needle placement more easily. This can be especially important in busy or resource‐limited settings or when used by less experienced anaesthetists seeking to reduce technical difficulty. Nonetheless, this convenience comes at the cost of a significantly increased risk of PDPH due to the greater dural disruption caused by the larger needle size.

SA administered by anaesthetists with less than 3 years of experience was associated with a 2.7‐fold higher risk of developing PDPH compared to those with more than 3 years of experience. This finding is consistent with studies conducted in Gondar and Dill, Ethiopia [13, 15]. In our study, patients who received SA from students or anaesthetists with less than 3 years of experience developed PDPH more frequently than those treated by more experienced practitioners. However, this result differs from other studies done in Nairobi, Kenya and Dre‐Daw, Ethiopia, which reported no significant association between the anaesthetist’s level of experience and the incidence of PDPH. This discrepancy is due to the fact that, in our study, most SA procedures performed by students develop PDPH. The data collection was entirely complete; this means the finding can be applied to the real‐world.

### 4.1. Limitation of the Study

This study did not include the amount of CSF leakage as a factor, as it was not documented in parturient records. However, the volume of CSF loss may influence the occurrence of PDPH.

## 5. Conclusion

The incidence of PDPH in this study is higher than that reported in studies conducted in Ethiopia, Middle Eastern, and Asian countries. This study identified key predictors of PDPH among mothers undergoing CS under SA; those are multiple attempts, large‐gauge spinal needles and professionals with less than 3 years of experience. These findings highlight the need for targeted interventions, such as improving training for less experienced providers, minimising the number of puncture attempts and utilising smaller gauge needles, to reduce the incidence and burden of PDPH in clinical practice.

## Author Contributions

Esubalew Tesfahun: conceptualization, methodology, investigation, software, supervision, formal analysis, validation, and writing–original draft. Eden Takele: conceptualization, methodology, investigation, software, supervision, formal analysis, validation, and review and editing manuscript. Dereje Andargie: software, methodology, data curation, analysis, supervision, and review and editing manuscript. Solomon Hailemeskel: validation, investigation, analysis, data curation, supervision, methodology, and review and editing manuscript.

## Funding

No funding was received for this research.

## Disclosure

All authors read and approved the final version of the manuscript.

## Ethics Statement

This manuscript provides an honest, accurate, and transparent account of the reported study. All essential aspects of the study have been included, and any deviations from the original plan (and, if applicable, the registered protocol) have been clearly explained.

## Conflicts of Interest

The authors declare no conflicts of interest.

## Data Availability

The data used in this study for analysis are available from the corresponding author upon reasonable request.
